# THE DUAL IMPACT OF EDUCATION AND OCCUPATION ON COGNITIVE FUNCTIONING IN OLDER MEXICAN ADULTS

**DOI:** 10.1093/geroni/igad104.3631

**Published:** 2023-12-21

**Authors:** Jose Cabrero Castro, Mariela Gutierrez, Theresa Andrasfay, Emma Aguila Vega, Brian Downer

**Affiliations:** University of Texas Medical Branch, Galveston, Texas, United States; University of Texas Medical Branch, Galveston, Texas, United States; California State University San Marcos, San Marcos, California, United States; University of Southern California, Los Angeles, California, United States; University of Texas Medical Branch, Galveston, Texas, United States

## Abstract

Our research investigates the relationships among educational attainment, occupational mental demands, and cognitive functioning in Mexican adults aged 50 and older. We expand on prior studies demonstrating education’s link to cognitive function by examining how the complexity of one’s primary occupation also influences cognitive health. We hypothesize that cognitively demanding work, irrespective of education level, correlates positively with enhanced cognitive performance. Furthermore, we propose an additive effect, wherein high occupational mental demands amplify cognitive functioning beyond education’s influence. Our study uses data from 12,939 participants in the 2012 Mexican Health and Aging Study (MHAS) linked to an occupational database (Occupational Information Network O*NET). Employing generalized linear models to predict a summary cognition score, the findings unveil compelling insights. Both mental processes (β=0.17) and cognitive abilities (β=0.16) required in participants’ main occupations demonstrate positive correlations with cognitive scores (p<.01). Education emerges as a predictor of cognitive performance and had a stronger relationship with cognitive functioning compared to occupational mental demands (β=0.79, p<.01), highlighting the long-lasting effects of education in cognition emphasizing its role in shaping cognitive abilities throughout life. Adjusting for demographic and health factors reaffirms these associations. Moreover, our research introduces an innovative approach by creating an additive score for education and occupational mental demands. Our results reveal that the combined impact of education and mental demands is associated with higher cognitive scores (β=0.97, p<.01). In conclusion, our study presents a novel perspective by demonstrating that a combination of education and occupational mental demands may yield amplified cognitive benefits.

